# Effectiveness and safety of Lacosamide therapy for children with focal epilepsy: a real world study

**DOI:** 10.3389/fphar.2023.1186768

**Published:** 2023-08-09

**Authors:** Chunsong Yang, Zheng Liu, Yuxuan Peng, Lingli Zhang, Dan Yu

**Affiliations:** ^1^ Department of Pharmacy, West China Second University Hospital, Sichuan University, Chengdu, China; ^2^ Evidence-Based Pharmacy Center, West China Second University Hospital, Sichuan University, Chengdu, China; ^3^ NMPA Key Laboratory for Technical Research on Drug Products in Vitro and in Vivo Correlation, Chengdu, China; ^4^ Key Laboratory of Birth Defects and Related Diseases of Women and Children, Sichuan University, Ministry of Education, Chengdu, China; ^5^ West China School of Medicine, Sichuan University, Chengdu, China; ^6^ West China School of Pharmacy, Sichuan University, Chengdu, China; ^7^ Chinese Evidence-based Medicine Center, West China Hospital, Sichuan University, Chengdu, China; ^8^ Department of Children’s Genetic Endocrinology and Metabolism, West China Second University Hospital, Sichuan University, Chengdu, China

**Keywords:** focal-onset epilepsy, lacosamide, children, effectiveness, safety

## Abstract

**Objectives:** To compare the effectiveness and safety of the new antiepileptic drug, lacosamide (LCM) with Levetiracetam, for the treatment of focal epilepsy in children.

**Methods:** This study was a cohort study. Children with focal epilepsy who received LCM or Levetiracetam treatment in West China Second Hospital of Sichuan University were recruited and followed up for 12 months. Changes in the frequency of epilepsy, 50% and 75% responder rates, and seizure freedom rates from baseline to the maintenance period and adherence score were assessed. In addition, adverse events (AEs) were recorded.

**Results:** 92 patients completed the study, and were divided into two groups: LCM (*n* = 46) and Levetiracetam (*n* = 46). Participants were aged from 2 to 16.3 years, with a mean epilepsy duration of 2.57 years. The average maintenance dose of LCM was 5.03 ± 1.91 mg/kg/d after the titration period. There was no significant difference between the two groups in terms of the mean seizure frequency during subsequent visits at 1, 3,6, 9, 12 months. There was significant difference between the two groups in terms of the 50% responder rate at 6 months. No serious AEs were reported in both groups. The vast majority of patients had good adherence (adherence score = 4) in the LCM group.

**Conclusion:** LCM is effective as adjunctive therapy in children with epilepsy and has good safety, tolerability and adherence. Large sample size studies with long-term follow-up are needed in the future to comprehensively evaluate the use of LCM in children.

**Clinical Trial Registration**: [https://www.chictr.org.cn/showproj.html?proj=41041], identifier [ChiCTR1900024507].

## 1 Introduction

Epilepsy is one of the most common chronic neurological diseases, affecting approximately 65 million people worldwide and about 1% of the US population. It has an incidence of 50–100 per 100,000 individuals per year, with higher incidence rates among those younger than 1 year and older than 85 years. (Beghi, 2020; Andres and Manuel, 2022). The first onset of epilepsy is generally during childhood and adolescence, the epidemiology of epilepsy in early childhood showed that the adjusted incidence of epilepsy presenting in the first 3 years of life was 239 per 100,000 live births and 36% of children had drug-resistant epilepsy, and 49% had global developmental delay at 24 months after presentation (Joseph et al., 2021). Because of the complex pathogenesis and the varied clinical manifestations of epilepsy in children, diagnosis and treatment can be challenging. According to the classifications of epilepsy by the International Antiepileptic Society, focal epilepsy is the most common type of epilepsy that accounts for more than 50% of epilepsy in children. (Falco-Walter, 2020).

Currently, the main treatment for epilepsy is drug therapy, and surgery is only considered when seizures cannot be controlled even with a combination of drugs. Although traditional antiseizure medications (ASMs) can partially control seizures, numerous children continue to experience poor control of seizures. More than 30% of children with epilepsy are insensitive to conventional ASMs and gradually develop refractory epilepsy. (Kwan et al., 2010; Verrotti et al., 2011). Traditional ASMs are often accompanied by various adverse events (AEs). Epilepsy has significant adverse effects on children both mentally and physically and places considerable economic burdens on medical insurance. The data for pediatric adverse reactions (ADRs) to ASM from the Italian spontaneous reporting system showed that the most commonly reported ADRs were skin rashes (24.0%), epileptic seizures (12.6%), gastrointestinal disturbances (11.8%), and somnolence (10.6%). Skin rashes were the most commonly reported ADR for lamotrigine (62.3%), carbamazepine (50.3%), phenobarbital (42.3%), and oxcarbazepine (33.0%). Other most commonly reported ADRs were gastrointestinal symptoms for ethosuximide (44%), irritability/aggression for levetiracetam (25.0%), epileptic seizures for valproic acid (16.1%), fever (often associated with hypohidrosis) for topiramate (17.9%), and utilization error (mostly accidental drug administration) for clonazepam (34.6%) (Franco et al., 2021). Therefore, there is an urgent need to develop new ASMs.

Lacosamide (LCM) was first approved by the United States Food and Drug Administration (FDA) and the European Medicines Agency (EMA) in 2008 as an adjunctive treatment for focal epilepsy in adults and adolescents. Then, it is approved in EMA as monotherapy in the treatment of partial-onset seizures with or without secondary generalisation in adults, adolescents and children from 2 years of age with epilepsy. In 2021, it is approved in FDA as for treatment of partial-onset seizures in patients 1 month of age and older and adjunctive therapy in the treatment of primary generalized tonic-clonic seizures in patients 4 years of age and older. In 2020, LCM was approved in China as an adjunctive therapy or monotherapy in children with focal epilepsy (aged over 4 years) (Wechsler et al., 2014; Baulac et al., 2017; Del Bianco et al., 2019; Yang et al., 2022) LCM is a novel N-methyl-D-aspartate receptor glycine site antagonist and is the only known ASM that selectively enhances the slow inactivation of voltage-gated sodium channels without affecting the rapid inactivation of sodium channels. LCM reduces the proportion of depolarized sodium channels to control epileptic seizures. (Rogawski et al., 2015). Compared with traditional ASMs, LCM has better pharmacokinetic properties: 100% oral bioavailability, less than 15% plasma-protein binding rate, and fewer drug interactions. In addition, LCM has little effect on liver and renal function in children; moreover, it has similar pharmacokinetics in both children (aged 4–16 years) and adults. (Hoy, 2018).

In 2022, Yang et al. (2022).conducted a meta-analysis to assess the effectiveness and safety of LCM in pediatric patients with epilepsy, and included 21 studies involving 1230 pediatric patients, they concluded that LCM is generally effective and well tolerated to use in children with epilepsy. However, further research with high-quality data and long-term follow-up of LCM use in pediatric populations is needed. Because this meta-analysis only included one RCT and two cohort studies, the majority of the included studies were case series studies, and the application of LCM in children with epilepsy in China has only recently been initiated.

In 2022, Tayla et al. (2022) conducted a retrospective cohort study to assess LCM as an add-on therapy for children with refractory epilepsy and found there was a reduction of >50% in the frequency of seizures in 73.1% of the children after 3 months treatment. Driessen et al., 2022 retrospectively evaluated the efficacy and tolerability of LCM in children with drug resistant epilepsy and concluded that LCM is an effective ASM with acceptable side-effects in children with drug-resistant epilepsy.

However, there are few studies involving the control group, and research data based on the Chinese child population is lacking. Therefore, more reliable clinical data for children from China are needed to evaluate its safety and effectiveness. It is necessary to conduct more clinical research to compare the effectiveness and safety of LCM with other ASMs. Real-world evidence helps to improve decision making in healthcare settings. Hence, this study aimed to analyze the effectiveness and safety of LCM for the treatment of epilepsy in children in a real-world setting.

## 2 Materials and methods

### 2.1 Study design

This was an open-label, non-interventional, post-marketing, observational study to investigate the effectiveness and safety of LCM for children with epilepsy in a real-world practice, which was registered in the Chinese Clinical Trial Registration Center (registration number: ChiCTR1900024507).

### 2.2 Participants

Patients who were diagnosed with focal epilepsy according to the 2017 International League Against Epilepsy (ILAE) Seizure Classification at West China Second Hospital, Sichuan University (Scheffer et al., 2017), from January 2020 to December 2021 were recruited to the LCM treatment group and Levetiracetam group.

The inclusion criteria were as follows: 1) patients or their legal representative provided written informed consent; 2) patients were aged younger than 18 years; 3) diagnosed with focal epilepsy following clinical examination; 4) treated with LCM as adjunctive therapy; 5) had at least one seizure in the year prior to starting LCM; 6) Their guardians are willing to attend the follow-up of medication treatment.

The exclusion criteria were as follows: 1) patients were aged over 18 years; 2) allergic to LCM; 3) patients with second- and third-degree atrioventricular block; 4) patients with liver or renal dysfunction (alanine aminotransferase (ALT) or qspartate aminotransferase (AST) of over two times the upper normal limit, urine creatinine (CR) over the upper normal limit); 5) patients who had no seizures in the year prior to starting LCM.

### 2.3 Observation and control group

All patients were treated with LCM as adjunctive therapy in addition to basic traditional antiepileptic therapy. LCM treatment was divided into titration (dosing period) and maintenance periods. Patients received an initial dose of LCM at the beginning, and after a titration period of 1–6 weeks, the LCM dose was gradually increased every 1–2 weeks to the maximum dose, which was maintained for at least 12 months, twice a day. The control group used Levetiracetam, the dose ranged from 10 to 30 mg/kg, twice a day.

### 2.4 Data collection

The follow-up duration was 12 months. Patients and their guardians were followed up at months 1, 3 6, and 12 of the LCM treatment maintenance periods through questionnaires and telephone follow-ups. Demographic, effectiveness, and safety data were collected, which included sex, age, weight, epilepsy duration, newly diagnosed patients (the patients were diagnosed with epilepsy within 3 months prior to participation in this study), family history of epilepsy (the patient has either parents or siblings with a diagnosis of epilepsy), number of concomitant ASMs at baseline, dosage of concomitant ASMs at baseline, initial dose of LCM, maintenance dose of LCM titration solution, seizure frequency.

### 2.5 Key outcomes

#### 2.5.1 Effectiveness outcomes

Effectiveness outcomes were: 1) seizure frequency every 28 days; 2) 50% responder rate: proportion of children with 50% or greater reduction in seizure frequency within 28 days; 3) 75% responder rate: proportion of children with 75% or greater reduction in seizure frequency within 28 days; 4) 100% responder rate: proportion of children with 100% or greater reduction in seizure frequency within 28 days. Patients with less than 50% reduction in seizure frequency were defined as “non-responders,” those with 50% or greater reduction in seizure frequency were defined as “responders,” those with 75% or greater reduction in seizure frequency were defined as “significant responders,” and those with 100% reduction in seizure frequency were defined as “seizure-free.”

#### 2.5.2 Safety outcomes

Safety outcomes were: 1) total incidence of AEs (TAE): ratio of all AEs to the total number of evaluable adverse events; 2) incidence of any AEs: ratio of the number of specific adverse events to the total number of evaluable adverse events; 3) incidence of serious adverse drug events (SAEs): SAEs are defined as events that require hospitalization, prolonged hospital stays, or disability or those that affect work ability, endanger life, cause death, or lead to congenital malformations during the clinical trial.

#### 2.5.3 Adherence outcomes

The Morisky Medication Adherence Scale (MMAS-4) was used to assess patients’ medication adherence. MMAS-4 consists of four questions with a scoring scheme of “Yes” = 0 and “No” = 1, and the items are summed to give a range of scores from 0 to 4. Higher scores indicate better adherence, scores of 4 were considered to show high adherence, scores of 0–3 low. We attended a training and certification session for the Morisky Widget in August 2019 in Beijing, China, and obtained licenses for the use of MMAS-4 from MMAS Research LLC, United States.

### 2.6 Sample size calculation

We used a comparison formula of two independent sample rates to conduct sample size calculation, the responder rate for LCM and control group was 65% vs. 38% [12], alpha = 0.05, beta = 0.20, the sample size for each group is 50.

### 2.7 Statistical methods

All experimental data were analyzed using SPSS version 22 (IBM, Armonk, NY, United States). For comparability of baseline variables with continuous variables, the Kolmogorov–Smirnov test was used for normality, data are shown as means ± standard deviations or median and interquartile range. T-tests or Mann–Whitney *U* test were used to evaluate the change in the number of seizures from baseline to after LCM or Levetiracetam treatment. Categorical variables are shown as frequencies and were analyzed using the chi-square or Fisher’s exact test as appropriate, and *p* < 0.05 was considered statistically significant.

### 2.8 Ethical Issues

The study was approved by the Office of Research Ethics Committees of West China Second Hospital. Written informed consent was obtained from patients or their legal representative, and consent was obtained from children aged >8 years, otherwise, the guardian signed the informed consent form.

## 3 Results

### 3.1 Demographic data

A total of 100 children with epilepsy from West China School University Hospital, Sichuan University were enrolled in the study, eight patients withdrew from this clinical study because they withdrew their consent and did not sign the informed consent, so 92 patients completed the study, these patients were divided into two intervention groups: LCM (*n* = 46) and Levetiracetam (*n* = 46) Table 1.

**TABLE 1:** The basic characteristics of patients.

	LCM (n = 46)	Levetiracetam (n = 46)	χ^2^/t	*P*
**Gender**				
Male	23 (50%)	22 (47.8%)	0.043	0.835
Female	23 (50%)	24 (52.2%)		
**Age**	8.50 ± 3.33	7.25 ± 3.16	1.845	0.068
**Weight**	30.04 ± 11.68	27.08 ± 12.15	1.192	0.237
**Newly diagnosed patient**				
Yes	7 (15.2%)	11 (23.9%)	1.105	0.293
No	39 (84.8%)	35 (76.1%)		
**Time of disorder**	2.22 ± 2.20	2.92 ± 2.77	−1.346	0.182
**Family history of epilepsy**			1.108	0.292
Yes	6 (13%)	3 (6.5%)		
No	40 (87%)	43 (93.5%)		
**Comorbidity**			1.373	0.241
Yes	10 (21.7%)	15 (32.6%)		
TD	6 (13%)	4 (8.7%)		
ADHD	2 (4.3%)	2 (4.3%)		
Growth and development disorder	1 (2.2%)	5 (10.9%)		
Sleep disorder	0	3 (6.5%)		
Hypophrenia	1 (2.2%)	1 (2.2%)		
No	36 (78.3%)	31 (67.4%)		

TD (tic disorder), ADHD (Attention deficit hyperactivity disorder), LCM (lacosamide).

Participants were aged from 2 to 16.3 years, with an average age of 7.8 ± 3.3. Epilepsy duration ranged from 0 to 11.4 years, with a mean duration of 2.57 ± 2.51. 19.6% (18/92) participants were newly diagnosed patient, 27.2% (25/92) of the patients had a history of other comorbidities.

The average initial dose of LCM was1.68 ± 0.57 mg/kg/d, and the average maintenance dose was 5.03 ± 1.91 mg/kg/d after the titration period. All patients were treated with LCM as adjunctive therapy in addition to other first-line ASMs (Levetiracetam = 17, Pirampanai = 13, Oxcarbazepine = 16). The average maintenance dose of Levetiracetam was 18.15 ± 5.20. There was no significant difference between the two groups in terms of gender, age, weight, newly diagnosed patient, time of disorder, family history of epilepsy, and comorbidity.

### 3.2 Effectiveness analysis

#### 3.2.1 100% responder rate

There was no significant difference between the two groups in terms of the mean seizure frequency during subsequent visits at 1, 3, 6, 12 months Table 2. The 100% responder rate of patients who received LCM or Levetiracetam was 47.8% vs. 52.2% (*p* = 0.677), 60.9% vs. 58.7% (*p* = 0.832), 67.4% vs. 58.7% (*p* = 0.388) and 71.7% vs. 60.9% (*p* = 0.270) at 1, 3, 6, 12 months.

**TABLE 2:** Effectiveness and adherence analysis in different treatment times.

Item		LCM (n = 46)	Levetiracetam (n = 46)	t/Z	*P*
		Mean ± SD or median (interquartile range)			
Seizure frequency at the time of enrollment		2 (2)	2 (3)	−1.952	0.051
The first month	Seizure frequency	1 (2)	0 (2)	−0.241	0.810
	Adherence score	4 (0)	4 (1)	−3.127	0.002*
	100% responder rate				
	Yes	22 (47.8%)	24 (52.2%)	0.174	0.677
	No	24 (52.2%)	22 (47.8%)		
	75% responder rate				
	Yes	27 (58.7%)	24 (52.2%)	0.396	0.529
	No	19 (41.3%)	22 (47.8%)		
	50% responder rate				
	Yes	39 (84.8%)	33 (71.7%)	2.30	0.129
	No	7 (15.2%)	13 (28.3%)		
The third month	Seizure frequency	0 (1)	0 (1)	−0.193	0.847
	Adherence score	4 (0)	4 (1)	−2.532	0.011*
	100% responder rate				
	Yes	28 (60.9%)	27 (58.7%)	0.045	0.832
	No	18 (39.1%)	19 (41.3%)		
	75% responder rate				
	Yes	34 (73.9%)	32 (69.6%)	0.214	0.643
	No	12 (26.1%)	14 (30.4%)		
	50% responder rate				
	Yes	43 (93.5%)	38 (82.6%)	2.581	0.108
	No	3 (6.5%)	8 (17.4%)		
The sixth month	Seizure frequency	0 (1)	0 (1.25)	−1.203	0.229
	Adherence score	4 (1)	4 (1)	−1.982	0.048*
	100% responder rate				
	Yes	31 (67.4%)	27 (58.7%)	0.746	0.388
	No	15 (32.6%)	19 (41.3%)		
	75% responder rate				
	Yes	37 (80.4%)	30 (65.2%)	2.691	0.101
	No	9 (19.6%)	16 (34.8%)		
	50% responder rate				
	Yes	43 (93.5%)	36 (78.3%)	4.389	0.036*
	No	3 (6.5%)	10 (21.7%)		
The twelfth month	Seizure frequency	0 (1)	0 (1)	−1.204	0.229
	Adherence score	4 (1)	3 (2)	−2.625	0.009*
	100% responder rate				
	Yes	33 (71.7%)	28 (60.9%)	1.216	0.270
	No	13 (28.3%)	18 (39.1%)		
	75% responder rate				
	Yes	40 (87%)	33 (71.7%)	3.250	0.071
	No	6 (13%)	13 (28.3%)		
	50% responder rate				
	Yes	44 (95.6%)	39 (84.8%)	3.079	0.079
	No	2 (4.3%)	7 (15.2%)		

LCM (lacosamide), * (*p* < 0.05).

#### 3.2.2 75% responder rate

The 75% responder rate of patients who received LCM or Levetiracetam was 58.7% vs. 52.2% (*p* = 0.529), 73.9% vs. 69.6% (*p* = 0.643), 80.4% vs. 65.2% (*p* = 0.101) and 87% vs. 71.7% (*p* = 0.071) at 1, 3, 6, 12 months.

#### 3.2.3 50% responder rate

The 50% responder rate of patients who received LCM or Levetiracetam was 84.8% vs. 71.7% (*p* = 0.129), 93.5% vs. 82.6% (*p* = 0.108), 93.5% vs. 78.3% (*p* = 0.036) and 95.6% vs. 84.8% (*p* = 0.079) at 1, 3, 6, 12 months. There was significant difference between the two groups in terms of the 50% responder rate at 6 months.

### 3.3 Safety analysis

15.2% (7/46) and 21.7% (10/46) of patients experienced treatment related AEs in LCM and Levetiracetam group respectively, these AEs were mild and could be tolerated by the patient, so no emergent treatment was not needed, no SAEs were reported during LCM or Levetiracetam treatment Table 3.

**TABLE 3:** The AEs of the two groups.

	LCM (n = 46)	Levetiracetam (n = 46)
Total number of people with AEs	7	10
Enuresis	1	0
Dizziness	2	0
Irritability	1	2
Somnolence	1	0
Growth retardation	1	0
Vomiting	1	1
Drowsiness	1	3
Rash	0	2
Cough	0	1
Anorexia	0	1
Nausea	0	1
Diarrhea	0	1
Total number of AEs	8	12

AEs (adverse events).

The most common AEs of LCM was enuresis (2.2%, 1/46), dizziness (2.2%, 1/46), irritability (2.2%, 1/46), somnolence (2.2%, 1/46), growth retardation (2.2%, 1/46), vomiting (2.2%, 1/46) and drowsiness (2.2%, 1/46). The most common AEs of Levetiracetam was drowsiness (6.5%, 2/46), rash (4.3%, 2/46), irritability (4.3%, 2/46), vomit (2.2%, 1/46), cough (2.2%, 1/46), anorexia (2.2%, 1/46), nausea (2.2%, 1/46) and diarrhea (2.2%, 1/46).

### 3.4 Adherence analysis

The adherence score of LCM treatment group was higher than that in Levetiracetam treatment group at 1, 3, 6, 12 months, and there was significant difference between the two groups, but with the extension of treatment time, the scores of adherences decreased Table 2.

## 4 Discussion

### 4.1 Main findings

Since LCM was approved by the FDA and EMA in 2008, numerous studies have demonstrated its clinical safety and effectiveness in patients with focal epilepsy. (Villanueva et al., 2012; McGinnis and Kessler, 2016; Sanmartí-Vilaplana and Díaz-Gómez, 2018; Del Bianco et al., 2019; Ferreira et al., 2019). It was not until 2018 that China officially approved LCM as adjunctive therapy for patients with focal epilepsy aged over 16 years; thus, additional clinical data are needed to evaluate the safety and effectiveness in China. We recruited a total of 92 children with epilepsy who received LCM or Levetiracetam and followed up patients for 12 months. Results showed that after 12 months of LCM treatment, seizure frequency decreased from 4.20 ± 8.05 to 0.39 ± 0.77, and the 50% responder rate, 75% responder rate, and seizure-free rate of LCM were 71.7%, 87%, and 95.7%, respectively. Our findings indicated that adjunctive LCM was efficacious in reducing seizure frequency in children with epilepsy. During LCM treatment, the TAE was 13%, with the most common AEs involving the nervous and digestive system. No SAEs were reported, the medication adherence in LCM group was better than that in Levetiracetam group in children with epilepsy, which may be related to the better tolerability of the observation group”.

### 4.2 Comparison with other studies

In 2022, Romão et al., 2022 conducted a retrospective cohort study in a tertiary healthcare facility in Brazil, which included 26 children with refractory epilepsy, and found the reduction of >50% in the frequency of seizures was 73.1% and 73.9% after 3 months and 9 months of LCM treatment, mild AEs were observed in very few children. In 2016, a large double-blind randomized controlled trial revealed that the average 4-week frequency of seizure episodes during the maintenance period with the use of 200 and 400 mg/day of LCM decreased by 29.4% and 39.6% in Chinese and Japanese adults, respectively, and the most common AEs were dizziness and drowsiness. (Hong et al., 2016). In 2020, Feng found that the 50% responder rate for LCM in the treatment of focal and generalized seizures for children with refractory epilepsy in China was 49% and 51%, respectively, and the incidence of TAEs was 12%, with common AEs including vertigo, diplopia, nausea and vomiting, abnormal ataxia, and blurred vision (Feng et al., 2020). Results on the safety of the above studies were consistent with our studies, the reported AEs were mild, however, the effectiveness results differed slightly. Compared with the previous studies, the responder rate for LCM and the percentage reduction in seizure frequency per 28 days were higher in our study, this may be because our subjects were not refractory epilepsy, and seizures were not very frequent (the number of seizure was less than 5 at baseline during 4 weeks) at the time of inclusion in the study. In addition, the above research included refractory epilepsy (Feng et al., 2020; Romão et al., 2022), so the effect of drug control may be different in different types of epilepsy. In addition, the duration of the epilepsy time were 4.8 ± 3.4 and 4.49 ± 2.34, respectively (Feng et al., 2020; Romão et al., 2022), which was longer than that in our study, so this may be also one of the reasons for the differences in effectiveness. There are few studies comparing LCM and Levetiracetam in the treatment of focal epilepsy in children. In addition, few studies assessed the adherence of LCM treatment, we found children with epilepsy receiving LCM treatment have better adherence, so our studies provided more evidence for the treatment for children with epilepsy.

### 4.3 Limitations and future research

The present study has several limitations. First, this was a cohort study that did not use randomized block, which may have led to confounding bias. Second, the follow-up of adherence was conducted mainly by telephone. Therefore, subjective factors of patients could not be eliminated, which may have affected the accuracy of follow-up. Third, the sample size of 92 children was from a single research center, to some extent, which limited the generalizability of the research results.

Fourth, MMAS-4 was a subjective method, our studies may overestimate or underestimate medication adherence for children with epilepsy, so multiple methods assessment could be used in the future, such as self-report questionnaires, structured interviews, therapeutic drug monitoring (TDM), electronic devices, and pick-up/refill rates (Al-Hassany et al., 2019). Future research on LCM should focus on two aspects. First, previous studies have demonstrated a relationship between effectiveness and dose of LCM treatment. Future studies should pay attention to the effectiveness and safety in children for different doses of LCM. Second, in addition to LCM as adjunctive therapy, studies on the effectiveness and safety of LCM as monotherapy for focal epilepsy are also of great importance.

## 5 Conclusion

LCM is effective as adjunctive therapy in children with epilepsy and has good safety, tolerability and adherence. Large sample size studies with long-term follow-up are needed in the future to comprehensively evaluate the use of LCM in children.

## Data Availability

The original contributions presented in the study are included in the article/Supplementary material, further inquiries can be directed to the corresponding authors.
